# Circ-HMGA2 (hsa_circ_0027446) promotes the metastasis and epithelial-mesenchymal transition of lung adenocarcinoma cells through the miR-1236-3p/ZEB1 axis

**DOI:** 10.1038/s41419-021-03601-2

**Published:** 2021-03-24

**Authors:** Zhongjian Yu, Xiongjie Zhu, Ying Li, Min Liang, Meijun Liu, Zhile Liu, Lingyu Qin, Xiaoran Wu, Kunpeng Du, Lu Liu, Yong Wang, Yanfang Zheng

**Affiliations:** 1 Medical Oncology Department, Affiliated Cancer Hospital and Institute of Guangzhou Medical University, Guangzhou, China; 2 Department of Oncology, Zhujiang Hospital of Southern Medical University, Guangzhou, China; 3 Department of Oncology, The Fifth Affiliated Hospital of Guangzhou Medical University, Guangzhou, China; 4 Department of Medical Oncology, First Affiliated Hospital of Nanchang University, Nanchang, China

**Keywords:** Non-small-cell lung cancer, Metastasis

## Abstract

Lung adenocarcinoma (LUAD) has high incidence and mortality rates worldwide; however, its detailed molecular pathology remains unclear. Although circRNAs have gradually been identified as molecules that are differentially expressed in tumors and play key roles in tumor progression, their role in LUAD is poorly understood. Through microarray analysis, we obtained the circRNA expression profile of LUAD and found that circ-HMGA2 (hsa_circ_0027446), a novel RNA, is highly expressed in LUAD. The high expression of circ-HMGA2 was further verified in 36 paired LUAD and adjacent normal tissues. Functionally, circ-HMGA2 promoted LUAD cell metastasis in vitro and in vivo. The luciferase reporter assay and FISH results showed that circ-HMGA2 interacts with miR-1236-3p and that miR-1236-3p interacts with ZEB1. In addition, miR-1236-3p was expressed at low levels in LUAD, inhibited LUAD cell metastasis, and suppressed the function of circ-HMGA2. ZEB1 is an EMT-promoting transcription factor. The PCR and WB analysis results showed that circ-HMGA2 promotes both ZEB1 expression and EMT. MiR-1236-3p had the opposite effect, reversing the promotive effect of circ-HMGA2 on EMT. In summary, circ-HMGA2 promotes LUAD cell metastasis through the miR-1236-3p/EMT axis, indicating that it could be a therapeutic target in LUAD.

## Introduction

Lung cancer is the leading cause of cancer death, and its prognosis remains poor worldwide^1^. Lung adenocarcinoma (LUAD) is the most common type of lung tumor, accounting for 40% of lung tumors^2^. The average survival rate of patients with LUAD is very low, ranging from 4 to 17%^3^. Although surgery and other treatments for LUAD have greatly improved in recent years, the 5-year relative survival rate of LUAD is still very low^4^. Tumor metastasis is the leading cause of death in most cancer patients, including those with LUAD^5,6^. Therefore, discovering the mechanism of LUAD metastasis to improve early treatment and prolong the survival time of patients is an urgent need.

The mechanism of tumor metastasis is complex and poorly understood^7^. Epithelial-mesenchymal transition (EMT), during which epithelial cells gradually lose cell adhesion, undergo alterations in the cytoskeletal composition, and acquire mesenchymal traits, is an important component of the metastatic process^8,9^. Recently, accumulating evidence has shown that EMT is closely related to LUAD metastasis^10^. Zinc finger E-box binding homeobox 1 (ZEB1) has been reported to be a transcription factor (TF) that induces EMT and the metastasis of cells in diverse types of cancers^11^. ZEB1 orchestrates EMT by suppressing the expression of epithelial genes and upregulating mesenchymal markers^12^. In LUAD, expression of ZEB1 is an early critical event in tumor metastasis, indicating a reduced therapeutic response and poor prognosis^13,14^. To enhance the understanding of the roles of ZEB1 and EMT in LUAD, more in-depth study is needed.

Circular RNA (circRNA), as a novel form of RNA, has recently received increasing attention in tumor research. CircRNAs are highly conserved and stable because of their closed-loop structure and lack of 5′–3′ polarity^15^. CircRNAs are abundantly and differentially expressed in various diseases or stages of cancer; thus, some have been considered potential diagnostic biomarkers^16^. In tumors, some circRNAs act as oncogenes, affecting the occurrence and development of tumors and thus providing new therapeutic targets for the future^17^. Since circRNAs were reported to act as miRNA sponges^18,19^, an increasing number of studies have shown that they affect tumor occurrence and progression by affecting the function of one or more miRNAs^20^. For example, circ_0026344 suppresses the metastasis of human colorectal cancer cells by sponging miR-183^21^, and the circRNA hsa_circ_0001178 facilitates the invasion and metastasis of colorectal cancer cells by sponging multiple miRNAs to upregulate ZEB1 expression^22^. In addition, circRNAs have been found to act by interacting with proteins, and some circRNAs can even be translated into proteins^23,24^. Recently, circ-SOX4 was reported to promote LUAD progression through the miR-1270/PLAGL2/WNT axis^25^. Although the functions of some circRNAs in LUAD have been elucidated, the functions of numerous circRNAs in LUAD have not been identified, and this topic deserves further exploration.

In this study, circ-HMGA2 (hsa_circ_0027446), a newly identified circRNA, was found to be significantly overexpressed in LUAD. Both in functional studies in cells and in animal experiments, circ-HMGA2 was found to promote the metastasis of LUAD cells. Furthermore, research showed that circ-HMGA2 promotes the metastasis of LUAD cells through the miR-1236-3p/EMT axis.

## Materials and methods

### Specimens

A total of 36 pairs of LUAD and adjacent normal tissue samples were provided by the Cancer Hospital Affiliated with Guangzhou Medical University. All tissues were analyzed by quantitative real-time PCR (qRT-PCR). The study protocol was approved by the Ethics Committee of the Cancer Hospital Affiliated with Guangzhou Medical University, and all patients provided informed consent before enrollment. Tissues were stored at −80 °C until use.

### CircRNA microarray hybridization and data analysis

Total RNA was digested with RNase R (Epicentre, USA) to remove linear RNAs and enrich circRNAs. Then, the enriched circRNAs were amplified and transcribed to fluorescent cRNA utilizing a random priming method (Arraystar Super RNA Labeling Kit; Arraystar; USA). The labeled cRNAs were hybridized to an Arraystar Human circRNA Array (8x15K, Arraystar). After washing the slides, the arrays were scanned with an Agilent G2505C scanner. Agilent Feature Extraction software (version 11.0.1.1) was used to analyze the acquired array images. Quantile normalization and subsequent data processing were performed using the limma package in R software. CircRNAs with statistically significant differential expression between the two groups were identified through volcano plot filtering. Differentially expressed circRNAs between two samples were identified through fold change filtering. Hierarchical clustering was performed to visualize the distinguishable circRNA expression patterns among samples.

### Total RNA extraction and quantitative real-time PCR (qRT-PCR)

Total RNA was extracted from frozen tissues and cells using RNAiso Plus (Takara, Japan) according to the manufacturer’s instructions. RNA was quantified using a NanoDrop 2000 (Thermo Fisher Scientific, USA). cDNA was synthesized using PrimeScript RT Master Mix (Takara, China). qPCR was performed with TB Green Premix Ex Taq II (Takara, China) with the following thermal cycling program: 95 °C for 30 s and 40 cycles at 95 °C for 5 s, 60 °C for 30 s, and a dissociation step. The 2^−ΔΔCt^ method was used to calculate the relative expression level of each gene. The primer sequence is synthesized by Sangon Biotech (Supporting Table S1).

### Cell culture

The human lung adenocarcinoma (LUAD) cell lines used in this study (H1299 and A549) were purchased from ATCC (USA). All cells were cultured in RPMI 1640 medium (Gibco, USA) supplemented with 10% fetal bovine serum (FBS; Gibco, USA). All cell lines were incubated at 37 °C in a humidified atmosphere containing 5% CO_2_.

### Evaluation of the circular character of circ-HMGA2

The circular character of circ-HMGA2 was demonstrated by Sanger sequencing. The circ-HMGA2 sequencing results were compared with the sequences provided by circBASE^26^. To prove the stability of circular RNA, circ-HMGA2 and HMGA2 were treated with RNase R (Epicentre, USA), and the content of both was assessed by PCR and agarose gel electrophoresis. In addition, LUAD cells were treated with actinomycin D, and the stability of circ-HMGA2 and HMGA2 was verified by RT-PCR.

### Cell transfection

Three siRNAs targeting the back-splice site of circ-HMGA2 and the negative control siRNA were designed and synthesized by RiboBio (Guangzhou, China). The target sequences of the si-circ-HMGA2 constructs were as follows: si-has-1, 5′-CCTAGGAAATGGGAACCAA-3′; si-circ-2, 5′-AAATGGGAACCAACCGGTG-3′; si-circ-3, 5′-ATGGGAACCAACCGGTGAG-3′. The circ-HMGA2 overexpression plasmid was synthesized by GeneChem (Shanghai, China). MiR-1236-3p mimics/inhibitors were designed by and purchased from RiboBio (Guangzhou, China). Before transfection, cells were cultured to 60-70% confluence in 6-well plates. Lipofectamine 3000 was used for transfection according to the manufacturer’s protocols.

### Migration and invasion assays

For the migration assay, RPMI 1640 medium containing 20% FBS was added to the lower chambers of a 24-well transwell plate, and transfected cells were resuspended in serum-free medium and seeded in the upper chambers (approximately 4 × 10^4^ cells in a 200 µl volume per chamber). After 24 h of co-culture, a portion of the cells had migrated through the membrane insert (8 µm pore size) from the upper chambers into the lower chambers. Cells in the lower chambers were fixed with 4% paraformaldehyde for 25 min, stained with 0.1% crystal violet for 25 min, and finally counted under a microscope (LEICA, Germany) at ×50 magnification. In contrast to the migration experiment, the invasion assay required the addition of Matrigel (Corning, USA) to the upper chambers and an incubation time of 48 h. All other steps were consistent with those in the migration assay.

### Wound healing assay

LUAD cells were seeded in 6-well plates. When the cells were confluent in the plate, a linear wound was created in the center of the plates with a 200 µl pipette tip. In addition, the serum concentration was changed from 10 to 1%. Wound healing was evaluated with a microscope (LEICA, USA) at 0, 24, and 48 h, and was quantified as follows: wound healing rate = width of wound closure/width of original wound × 100%.

### Animal study

The animal study was authorized by the Animal Ethics Committee of Zhujiang Hospital of Southern Medical University. Female BALB/c nude mice were purchased from Guangdong Experimental Animal Center (Guangzhou, China). A lung metastasis model was randomly established by injecting 2 × 10^6^ H1299 cells into 4-week-old nude mice via the tail vein (*n* = 5 mice per group). Five weeks after injection, the mice were sacrificed. Their lungs were removed and subjected to hematoxylin and eosin (HE) staining. The lung metastatic nodules were carefully counted.

### Luciferase reporter assay

Before co-transfection, 293T cells were seeded in 96-well plates at a density of 1 × 10^4^ cells per well for 24 h. Lipofectamine 2000 (Invitrogen, USA) was used to transfect cells with the indicated transcript. Twenty-four hours after transfection, cells were lysed, and luciferase activity was analyzed by a dual-luciferase reporter assay system (Promega, USA).

### Fluorescence in situ hybridization (FISH)

The FISH probe was designed by RiboBio (Guangzhou, China). For the fluorescent probes, Cy3 was used as the marker for circ-HMGA2, and FAM was used as the marker for miR-1236-3p. 4′,6-Diamidino-2-phenylindole (DAPI) was used to stain nuclei. FISH was performed according to the instructions of the FISH kit (GenePharma, China). The stained cells were then visualized using a confocal microscope (Leica, Germany).

### Western blot (WB) analysis

RIPA buffer was added to cells and incubated with shaking on ice for more than half an hour. The lysate supernatant was collected, and a protease inhibitor (1%; ComWin Biotech, Beijing, China) was added. The supernatant was centrifuged, and the protein concentration was measured using a BCA Protein Assay Kit (Beyotime, Shanghai, China). Proteins were separated by 10% SDS-PAGE and were then transferred to PVDF membranes (Millipore, USA). After blocking for 1 h with 5% skim milk powder, membranes were incubated with the primary antibody at 4 °C overnight. The following primary antibodies were used: anti-ZEB1 (1:1000 dilution,; Proteintech, USA), anti-E-cadherin (1:1000 dilution, Cell Signaling Technology (CST), USA), anti-N-cadherin (1:1000 dilution; CST, USA), anti-vimentin (1:1000 dilution; Bioss, China), and anti-GAPDH (1:5000 dilution; Bioss, China). Membranes were washed three times for 10 min each in TBST containing 0.1% Tween and were then incubated with goat anti-rabbit or goat anti-mouse secondary antibodies (1:8000 dilution, Bioss, China) for 1 h at room temperature. Membranes were washed three times with TBST containing 0.1% Tween. Protein bands were detected with SuperSignal West Femto Agent (Millipore) and visualized with the Chemical Mp Imaging System (Bio-Rad).

### Immunohistochemistry (IHC)

IHC was performed as described previously^27^. Serial sections of tissue were incubated with antibodies specific for EMT-related proteins at 4 °C overnight and then with secondary antibodies for 1 h at room temperature.

### Statistical analyses

The student’s *t*-test was performed with SPSS 19.0 software to determine whether the differences between the two experimental groups were significant. *P* < 0.05 was used as the criterion for significant differences.

## Results

### Differentially expressed circRNAs in LUAD and adjacent normal tissues

**Fig. 1:**
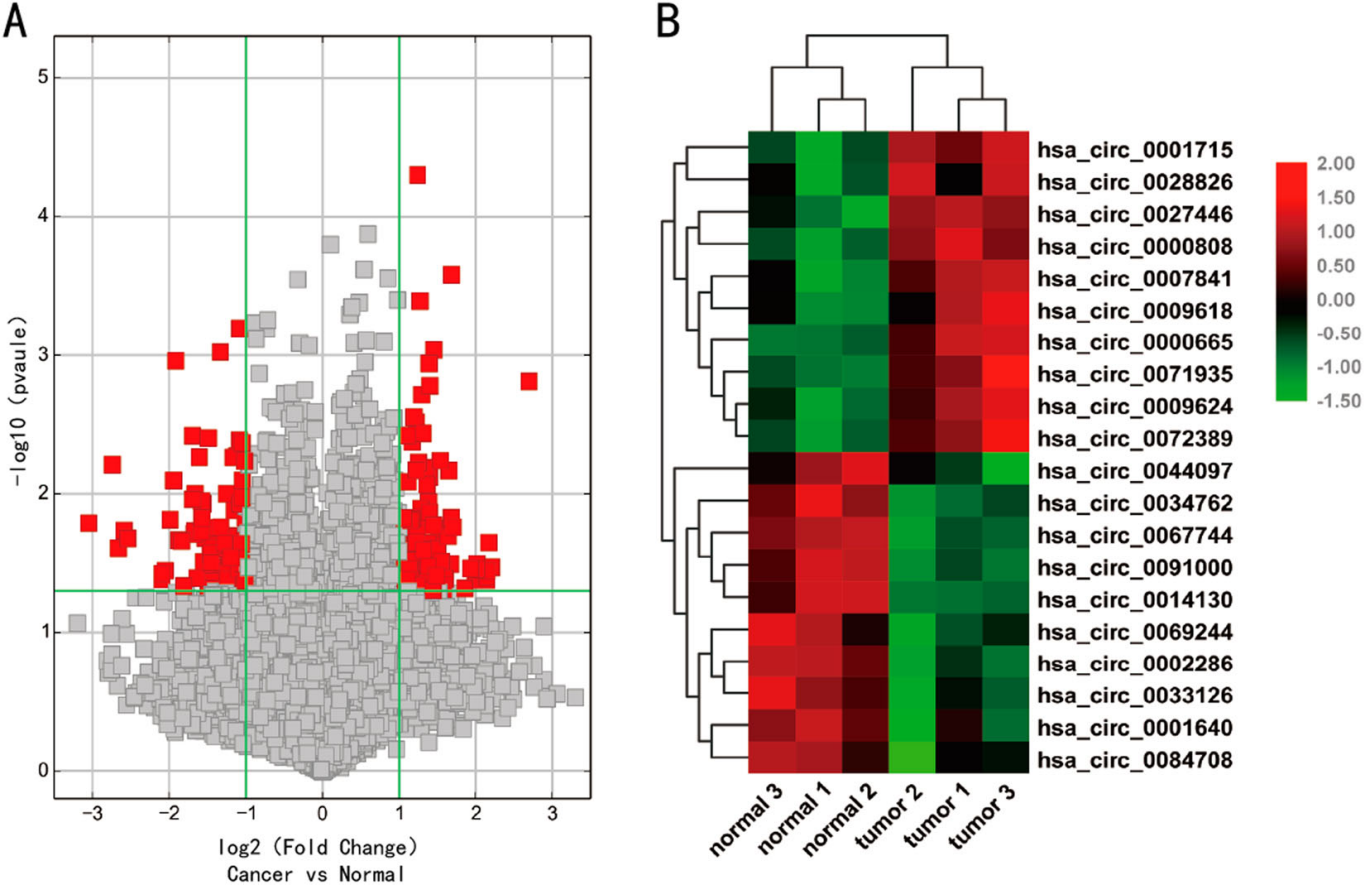
Differentially expressed circRNAs in LUAD tissues compared to adjacent normal tissues. **A** According to the criteria of fold change >2.0 and *P* < 0.05, the significantly aberrantly expressed circRNAs were selected from the volcano plot. **B** The heat map shows the top 10 circRNAs with the greatest increases and decreases in expression in 3 LUAD tissues compared to the paired adjacent normal tissues.

To filter the differentially expressed circRNAs in LUAD tissues and adjacent normal tissues, we performed a high-throughput human circular RNA microarray analysis of three pairs of LUAD and adjacent normal tissues. The analysis identified 12,444 distinct circRNAs, among which 85.7% were derived from exons, and the rest were derived from sense overlapping, intergenic, intronic, and antisense transcripts (Supporting Fig. S1A). A fold change of >2.0 and a *P* value of <0.05 were used as effective criteria to select significantly differentially expressed circRNAs. A volcano plot was constructed to visualize the differential expression patterns between tumor and nontumor tissues (Fig. 1A). It shows that 184 circRNAs were significantly aberrantly expressed in the circRNA microarray. Among these circRNAs, 103 were significantly upregulated and 81 were significantly downregulated in LUAD tissues compared with the paired adjacent normal tissues. In addition, the analysis identified 150 overlapping circRNAs and 30 novel circRNAs with respect to circBase^26^.

The hierarchical clustering results showed a distinguishable circRNA expression profile among the samples (Supporting Fig. S1B). To further narrow the field of circRNAs, we selected 10 of the most strongly upregulated and downregulated circRNAs for the preliminary experiment (Fig. 1B). Eventually, we found that hsa_circ_0027446 was related to LUAD metastasis.

### Characterization of hsa_circ_0027446 (circ-HMGA2) in LUAD

**Fig. 2:**
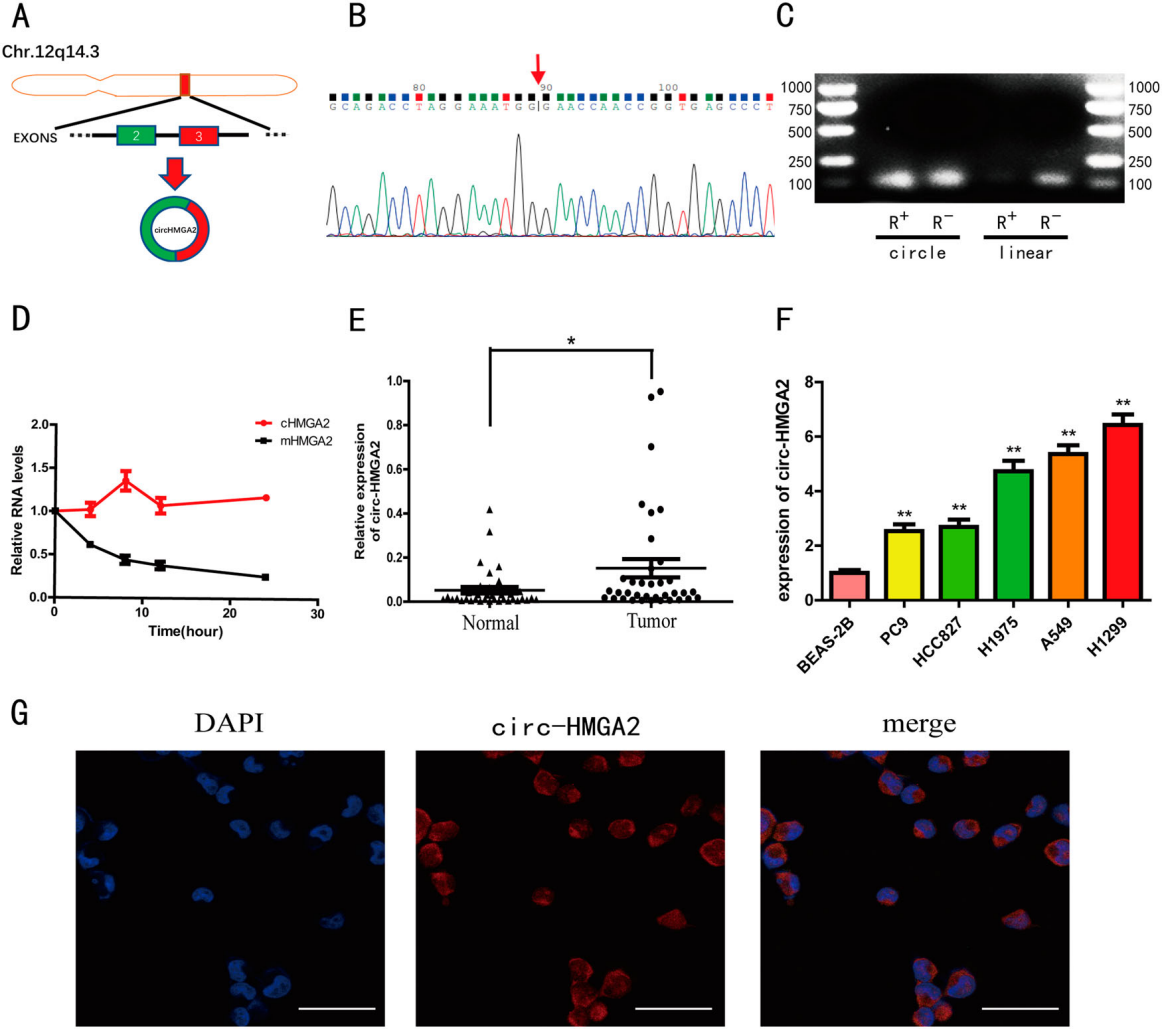
Characteristics of circ‐HMGA2 in LUAD cells. **A** The schematic illustration showing that circ-HMGA2 is cyclized from exons 2-3 of HMGA2. **B** Sanger sequencing showed the back-splice junction sites in circ-HMGA2. **C** Gel electrophoresis was performed to analyze RNase R digestion. Circ‐HMGA2 was resistant to RNase R digestion. **D** Upon exposure to actinomycin D, circ-HMGA2 was more stable than its linear form. **E** The relative expression levels of circ-HMGA2 in LUAD tissues and adjacent normal tissues were determined by qRT-PCR. **F** Circ-HMGA2 was significantly highly expressed in LUAD cells. **G** Circ-HMGA2 was localized mainly in the cytoplasm of H1299 cells, as verified by FISH. Scale bar: 25 μm. *P < 0.05.

Circ-HMGA2, located on chromosome 12q14.3, is derived from exons 2–3 of the HMGA2 gene through back-splicing (Fig. 2A). To prove its circular structure, the PCR products of circ-HMGA2 were analyzed by Sanger sequencing. The sequencing results were consistent with the sequence of the back-spliced region in circBase (Fig. 2B). Due to its circular structure, circRNA is more stable and resistant to RNase R digestion than linear RNA. The RNase R digestion experiment showed that the linear transcripts of HMGA2 were degraded, while the circular transcripts of HMGA2 were retained (Fig. 2C). In addition, treatment with actinomycin D demonstrated that circ-HMGA2 was more stable than the linear HMGA2 transcript (Fig. 2D). Moreover, the expression of circ-HMGA2 was significantly higher in the 36 LUAD tissues than in the paired adjacent normal tissues (Fig. 2E). Compared with BEAS-2B cells, circ-HMGA2 was also significantly highly expressed in five LUAD cells (Fig. 2F). The FISH results revealed that circ-HMGA2 was localized mainly in the cytoplasm (Fig. 2G). Taken together, these data showed the basic characteristics of circ-HMGA2 in LUAD cells.

### Circ-HMGA2 promotes LUAD cell metastasis by inducing EMT

**Fig. 3:**
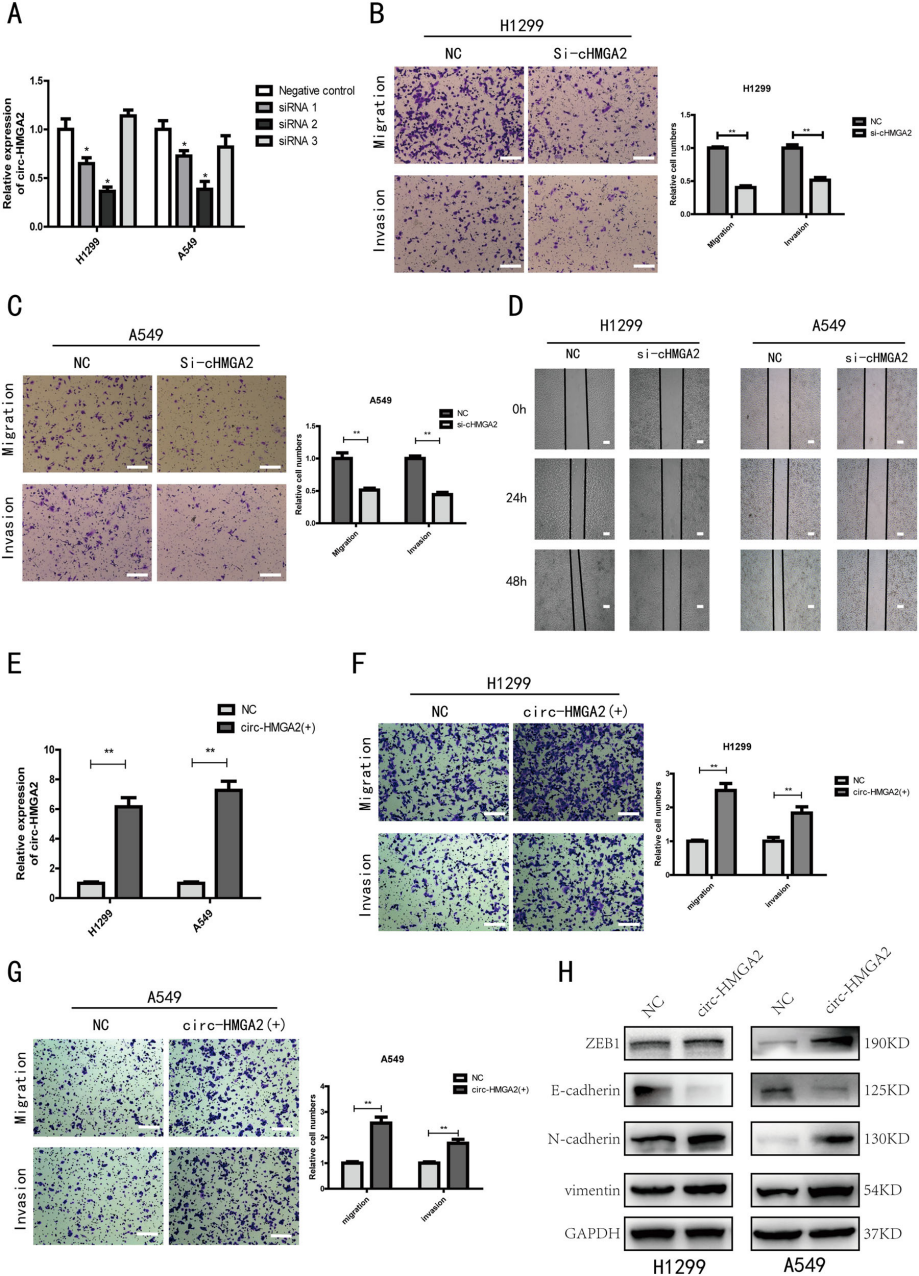
Circ‐HMGA2 promotes the metastasis of LUAD cells in vitro. **A** The expression of circ-HMGA2 in H1299 and A549 cell lines was significantly reduced after transfection with siRNA. **B**, **C** The results of transwell assays showed that the metastatic ability of H1299 and A549 cells was decreased after the circ‐HMGA2 knockdown. Scale bar: 200 μm. **D** The results of wound healing experiments showed that the migration of H1299 and A549 cells was decreased after circ‐HMGA2. Scale bar: 200 μm. **E** Circ-HMGA2 was significantly overexpressed in H1299 and A549 cells. **F**, **G** The metastatic ability was increased when the expression of circ-HMGA2 was enhanced. Scale bar: 200 μm. **H** Circ-HMGA2 had a promotive effect on EMT. *P < 0.05, **P < 0.01.

**Fig. 4:**
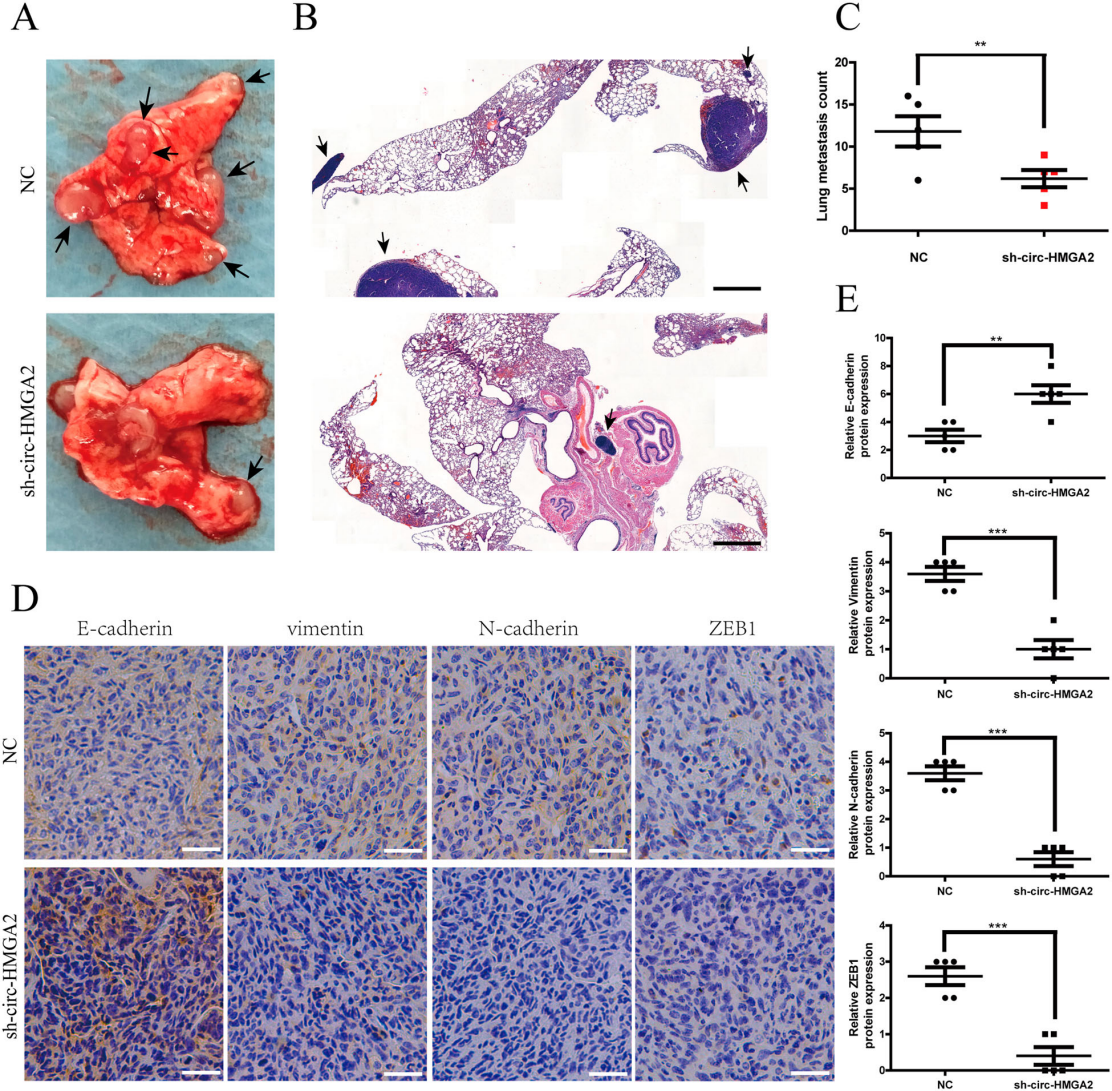
Circ‐HMGA2 promotes the metastasis of LUAD cells in vivo. **A** In the lung metastasis model, knocking down the expression of circ-HMGA2 resulted in a significant reduction in the number of metastases on the lung surface. **B**, **C** HE staining showed that mice in the circ-HMGA2 knockdown group showed significantly fewer metastases. Scale bar: 500 μm. **D**, **E** The results of IHC showed that knocking down the expression of circ-HMGA2 also inhibited the progress of EMT in vivo. Scale bar: 30 μm. **P < 0.01.

To investigate the biological function of circ-HMGA2 in LUAD, we knocked down the expression of circ-HMGA2 in H1299 and A549 cells. The qRT-PCR results showed that the transfection was successful (Fig. 3A). However, knockdown of circ-HMGA2 expression did not affect the expression of the linear HMGA2 transcript (Supporting Fig. S2). The results of the transwell assay showed that the migration and invasion abilities of H1299 and A549 cells were significantly reduced when the expression of circ-HMGA2 was inhibited (Fig. 3B, C). The wound healing experiments led to the same conclusion (Fig. 4D). But the proliferation of the cells has not been significantly affected (Supporting Fig. S3A). Similarly, we constructed a circ-HMGA2 overexpression vector and verified a significant increase in circ-HMGA2 expression by qRT-PCR (Fig. 3E). The migration and invasion abilities of H1299 and A549 cells with circ-HMGA2 overexpression were also enhanced, as shown by the results of the corresponding transwell assays (Fig. 3F, G).

EMT is an important phenotype in tumor metastasis. Therefore, we investigated whether circ-HMGA2 affects EMT in LUAD cells. WB analysis showed that overexpression of circ-HMGA2 in LUAD cells decreased the expression of the epithelial marker E-cadherin and increased the expression of the mesenchymal markers Vimentin and N-cadherin (Fig. 3H). It has the opposite result when knocking down the expression of circ-HMGA2 in the cells (Supporting Fig. S3B). These findings indicated that circ-HMGA2 promotes LUAD cell metastasis by inducing EMT.

### Circ-HMGA2 facilitates the metastasis of LUAD cells in vivo

To validate whether circ-HMGA2 positively affects metastasis in vivo, we established lung metastasis models by intravenously injecting H1299 cells into nude mice. After six weeks, lung colonization was evaluated. The lung metastasis model showed that the number of metastatic nodules on the lung surface of the knockdown circ-HMGA2 group was significantly less than that of the control group. (Fig. 4A). The HE staining results further verified knocking down the expression of circ-HMGA2 inhibits the metastasis ability of H1299 cells in vivo (Fig. 4B, C). In addition, the IHC results showed that inhibiting the expression of circ-HMGA2 can inhibit the process of EMT in tumor tissues (Fig. 4D, E). These results were consistent with our findings in vitro.

### Circ-HMGA2 acts as a sponge for miR-1236-3P

**Fig. 5:**
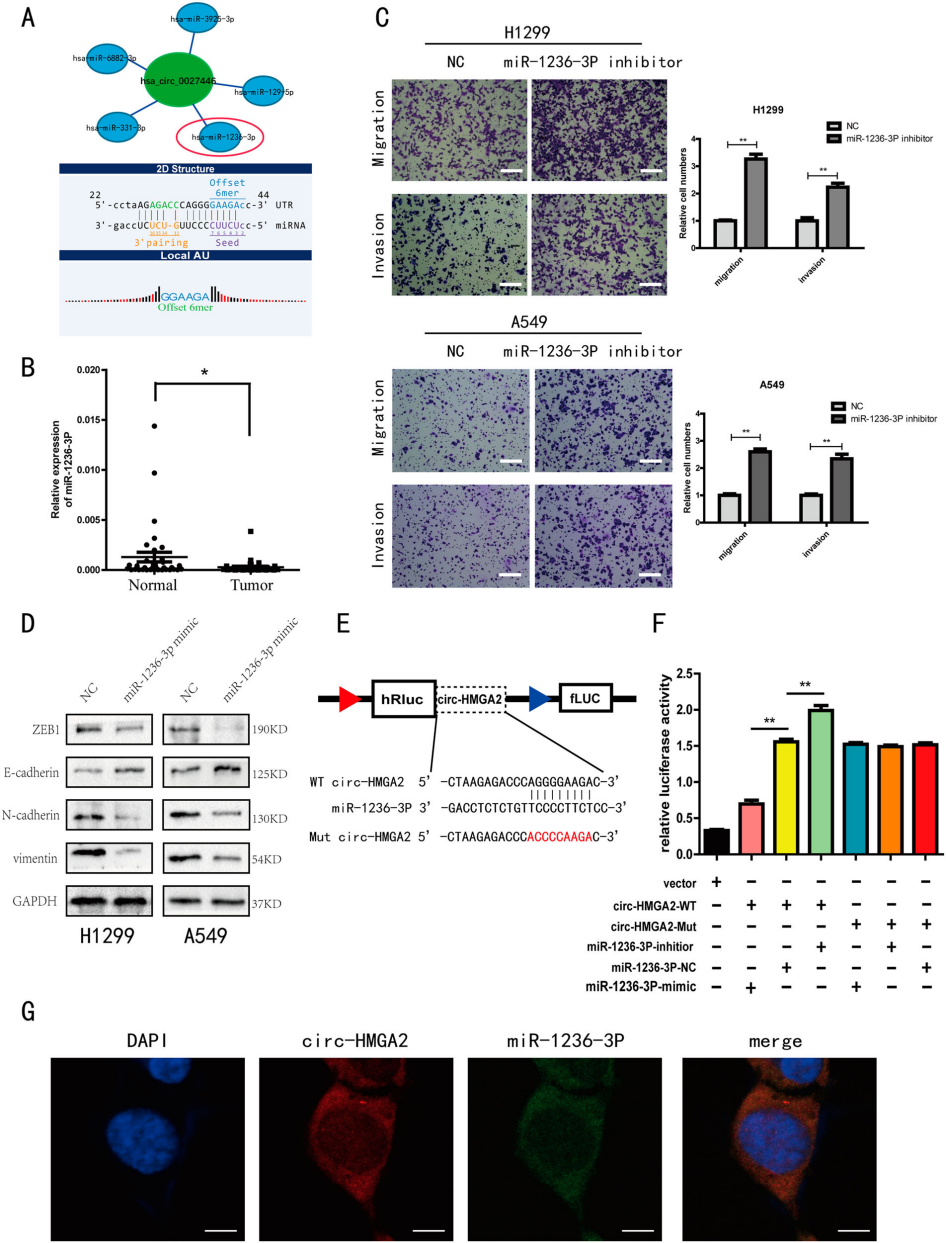
Circ-HMGA2 acts as a sponge for miR-1236-3p. **A** Analysis of the miRanda database indicated that circ-HMGA2 contains a binding site for miR-1236-3p. **B** The relative expression levels of miR-1236-3p in LUAD tissues and adjacent normal tissues were determined by qRT-PCR. **C** Transwell assays were performed in H1299 and A549 cells after transfection of the miR-1236-3p inhibitor. Scale bar: 200 μm. **D** miR-1236-3p inhibited EMT progression in LUAD cells. **E** Schematic illustration of circ-HMGA2 luciferase reporter vectors containing the wild-type (WT) or mutant (Mut) putative miR-1236-3p binding sites. **F** Luciferase activity was analyzed in 293T cells after co-transfection with miR-1236-3p mimics, miR-NC or miR-1236-3p inhibitors, and the WT or Mut luciferase reporter vectors. The relative luciferase activity was presented by the relative hRluc/hluc ratio. **G** The FISH results showed that circ-HMGA2 and miR-1236-3p are co-localized in the cytoplasm. Scale bar: 8 μm. *P < 0.05, **P < 0.01.

We found that circ-HMGA2 was composed of exons and was localized mainly in the cytoplasm. These results suggested that circ-HMGA2 may function by acting as a miRNA sponge^28^. To investigate miRNAs potentially involved in the function of circ-HMGA2, we used the miRanda database to identify the five miRNAs most likely to bind to circ-HMGA2 (Fig. 5A). The qRT-PCR data showed that miR-1236-3p expression was significantly lower in the 36 LUAD specimens than in the paired adjacent normal tissues (Fig. 5B). A prior study showed that miR-1236-3p can influence cancer metastasis^29^. We experimentally verified that miR-1236-3p inhibits the metastasis of LUAD cells (Fig. 5C). It also found that miR-1236-3p inhibits the progression of EMT (Fig. 5D). These results suggested that circ-HMGA2 may function by sponging miR-1236-3p. For the luciferase reporter assay, two luciferase reporter vectors containing circ-HMGA2 with wild-type and mutated miR-1236-3p binding sites were constructed (Fig. 5E). The luciferase reporter assay results showed that luciferase activity was negatively correlated with the expression of miR-1236-3p in cells transfected with the luciferase reporter construct containing wild-type circ-HMGA2. However, luciferase activity did not change significantly in cells transfected with the luciferase reporter construct containing mutant circ-HMGA2 was transfected into cells (Fig. 5F). Furthermore, the FISH results showed that miR-1236-3p was also localized mainly in the cytoplasm (Fig. 5G). Overall, these results indicated that circ-HMGA2 acts as a sponge for miR-1236-3p.

### Circ-HMGA2 inhibits LUAD metastasis through the circ-HMGA2/miR-1236-3p/EMT axis

**Fig. 6:**
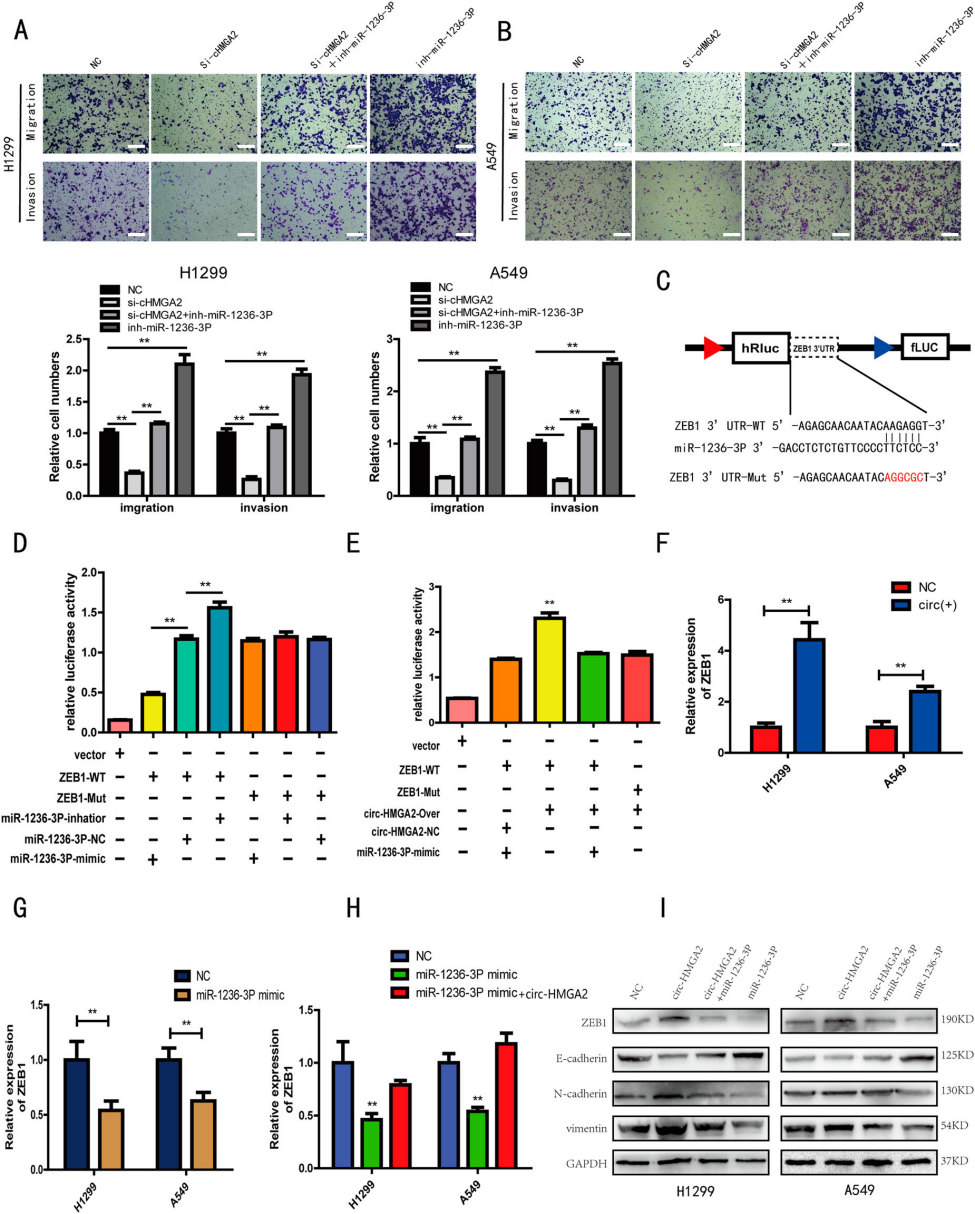
The circ-HMGA2/miR-1236-3p/EMT axis regulates LUAD cell metastasis. **A**, **B** The results of transwell assays showed that miR-1236-3p inhibited the promotive effect of circ-HMGA2 on LUAD metastasis. Scale bar: 200 μm. **C** Schematic of luciferase reporter vectors containing wild-type (WT) and mutant (Mut) ZEB1. **D** Luciferase activity was analyzed in 293T cells after co-transfection with miR-1236-3p mimics, miR-NC or miR-1236-3p inhibitors, and the WT or Mut luciferase reporter vectors. **E** Luciferase activity was analyzed in 293T cells after co-transfection with miR-1236-3p mimics, circ-HMGA2-NC or circ-HMGA2-over, and the WT or Mut luciferase reporter vectors. The relative luciferase activity was presented by the relative hRluc/hluc ratio. **F** The qRT-PCR results showed that circ-HMGA2 significantly increased the expression of ZEB1. **G** The qRT-PCR results showed that miR-1236-3p significantly inhibited the expression of ZEB1. **H**, **I** The mRNA and protein levels of ZEB1 and EMT was not changed after co-transfection with miR-1236-3p mimics and circ-HMGA2. **P < 0.01.

We found that circ-HMGA2 and miR-1236-3p are involved in the metastasis of LUAD and that circ-HMGA2 sponges miR-1236-3p. However, whether circ-HMGA2 acts through miR-1236-3p in LUAD required further investigation. Our experimental results demonstrated that the miR-1236-3p mimic inhibited the effect of circ-HMGA2 on LUAD metastasis (Fig. 6A, B). Via miRanda, we predicted that ZEB1 is downstream of miR-1236-3p. ZEB1 has been reported to be a transcription factor that induces EMT; thus, circ-HMGA2 may eventually lead to the metastasis of LUAD cells through promoting EMT^30^. To investigate the relationship between miR-1236-3p and ZEB1, two luciferase reporter vectors containing ZEB1 with wild-type and mutated miR-1236-3p binding sites were constructed(Fig. 6C). The luciferase reporter assay results showed that co-transfection of miR-1236-3p mimics and wild-type ZEB1 but not co-transfection of miR-1236-3p mimics and mutant ZEB1 significantly affected luciferase activity (Fig. 6D). Moreover, overexpression of circ-HMGA2 increased luciferase activity, but co-transfection of circ-HMGA2 and miR-1236-3p did not induce a change in luciferase activity (Fig. 6E). Next, overexpression of circ-HMGA2 was found to increase the expression of ZEB1 mRNA and protein and to promote EMT (Fig. 6F, Fig. 3H). However, transfection of miR-1236-3p mimics inhibited the ZEB1 expression and suppressed EMT (Fig. 6G, Fig. 4C). In addition, circ-HMGA2 blocked the effects of miR-1236-3p mimics on ZEB1 and EMT (Fig. 6H, I). It was also blocked the effects of si-ZEB1 on EMT (Supporting Fig. S4). In summary, these results indicated that circ-HMGA2 promotes the metastasis of LUAD cells by inhibiting the suppressive effect of miR-1236-3p on EMT.

## Discussion

Recently, accumulating evidence has shown that circRNAs are related to tumor occurrence and development and could have a future use as tumor biomarkers^20^. Some studies have shown that circRNAs play an important role in lung cancer. For example, upregulation of circ_0016760 promotes NSCLC cell progression through the miR-1287/GAGE1 axis^31^. Silencing of circ_0067934 suppresses the progression of NSCLC cells and correlates with unfavorable prognosis in NSCLC^32^. However, few studies on circRNAs in LUAD have been conducted. Currently, circ-HMGA2 was a novel circRNA never been reported before. Our study investigated circ-HMGA2 for the first time and its function in LUAD. We found that circ-HMGA2 was significantly highly expressed in LUAD through microarray, and its high expression was again verified by RT-PCR in LUAD cells and 36 pairs of clinical samples. In addition, its downstream miR-1236-3P has been reported to be related to the metastasis of a variety of cancers, and we verified its significantly low expression in LUAD tissues by RT-PCR. These suggest that the expression of circ-HMGA2 and miR-1236-3P may have important significance in the diagnosis of LUAD and may become biomarkers of LUAD in the future. This also implies that circ-HMGA2 has a potential role in LUAD.

Current research shows that circRNA is not simply a non-functional product of pre-mRNA splicing, as historically believed, and numerous circRNAs have been reported to play important roles in various diseases^33^. Especially in the occurrence and development of tumors, differentially expressed circRNAs can often act as oncogenes^34^. Our research proved that circ-HMGA2 can promote the metastasis of LUAD and the progression of EMT through in vivo and in vitro functional experiments. This is the first report of the function of circ-HMGA2 in LUAD. These results suggest that circ-HMGA2 may play an important role in assessing the progression of LUAD and provide a basis for becoming a potential therapeutic target in the future.

Previous reports indicate that circRNAs can act as miRNA sponges, thereby inhibiting the effects of miRNAs on their targeted mRNAs^35^. Through in-depth research, we found that circ-HMGA2 acts as a miRNA sponge to regulate LUAD metastasis. Bioinformatic analysis predicted that circ-HMGA2 binds to miR-1236-3p, and our study confirmed that circ-HMGA2 binds to miR-1236-3p and showed that both are localized in the cytoplasm. Substantial evidence indicates that miR-1236-3p can inhibit tumor metastasis in colorectal cancer, glioma, and gastric cancer^29,36,37^. Our research confirmed that miR-1236-3p can suppress metastasis and EMT and can inhibit the effect of circ-HMGA2 in LUAD. This study shows for the first time that circ-HMGA2 and miR-1236-3P are combined, and circ-HMGA2 promotes LUAD metastasis through miR-1236-3P. These results also suggest that miR-1236-3P plays an important role in the metastasis of LUAD and may become a potential therapeutic target in the future.

In addition, we found that miR-1236-3p binds ZEB1. ZEB1 is a well-known transcription factor that plays an important role in the metastasis of LUAD cells^38^. The relationship between ZEB1 and epigenetic regulation of EMT genes was discovered only recently. ZEB1 promotes epigenetic silencing of E-cadherin by recruiting multiple chromatin remodeling enzymes from the E-cadherin promoter^39^. Therefore, ZEB1 promotes the progress of EMT. Our study found that miR-1236-3p inhibits the expression of ZEB1, circ-HMGA2 promotes the expression of ZEB1, and miR-1236-3P would inhibit the promotive effect of circ-HMGA2 on ZEB1 expression. Further research showed that upon mutation of the miR-1236-3p binding site in circ-HMGA2, circ-HMGA2 no longer affected the expression of ZEB1. These results indicate that circ-HMGA2 acts as a sponge for miR-1236-3p to regulate the ZEB1/EMT pathway and promote LUAD cell metastasis. These also emphasize the important functions of circ-HMGA2 and miR-1236-3P in LUAD metastasis.

## Conclusions

**Fig. 7:**
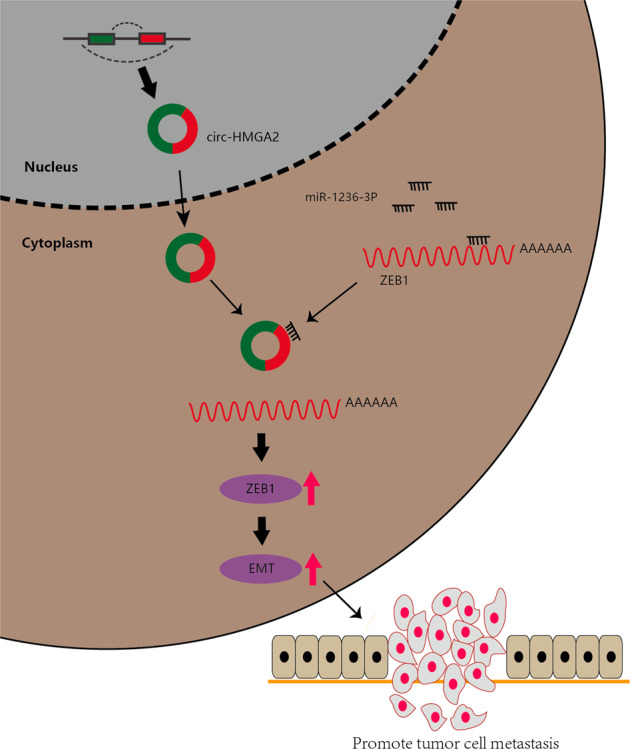
The circ-HMGA2/miR-1236-3p/ZEB1/EMT axis regulates LUAD cell metastasis. Circ-HMGA2 is highly expressed in lung adenocarcinoma and acts as a sponge of miR-1236-3P to promote the expression of ZEB1. ZEB1 acts as a transcription factor of EMT, promoting the progression of EMT and the metastasis of lung adenocarcinoma.

Our study provides the first evidence that circ-HMGA2 is highly expressed in patients with LUAD and promotes LUAD cell metastasis and EMT in vivo and in vitro. Mechanistically, the circ-HMGA2/miR-1236-3p/ZEB1 axis promotes EMT and metastasis of LUAD cells (Fig. 7). These results indicate that circ-HMGA2 could be a biomarker and an important therapeutic target in LUAD.

## Supplementary information

Figure S1

Figure S2

Figure S3

Figure S4

Supplementary Figure Legends
