# Supplementary figures and images for: Circ-HMGA2 (hsa_circ_0027446) promotes the metastasis and epithelial-mesenchymal transition of lung adenocarcinoma cells through the miR-1236-3p/ZEB1 axis
Source: Cell Death Dis. 2021 Mar 24;12(4):313. doi: 10.1038/s41419-021-03601-2 (PMC7991034; doi:10.1038/s41419-021-03601-2)

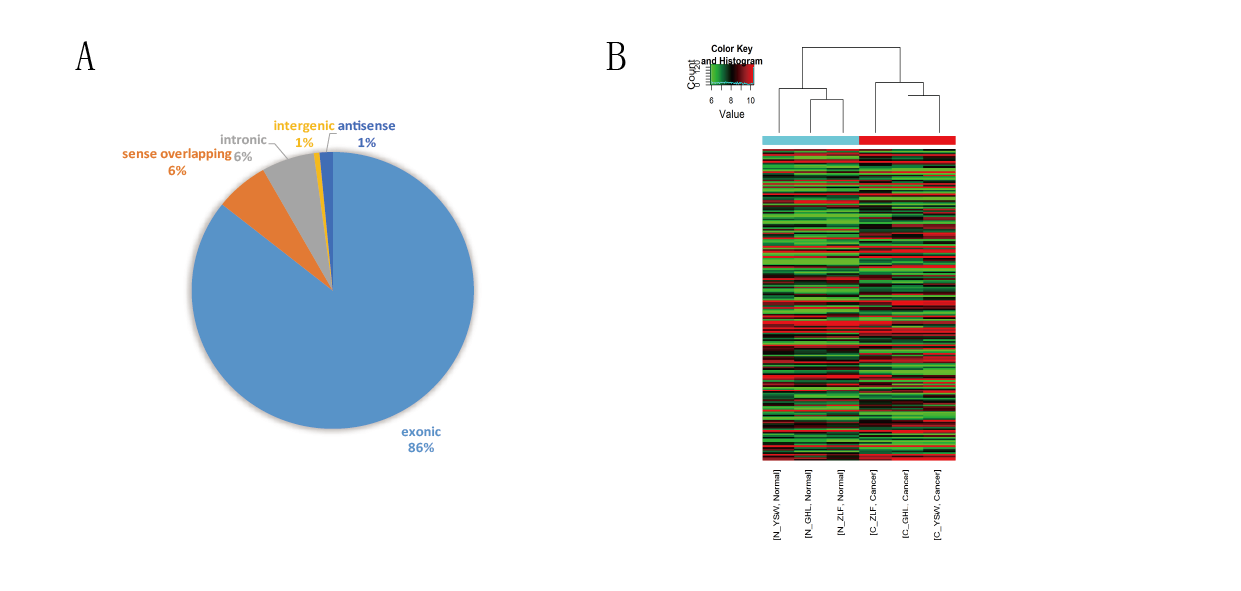

Supplement: Supplementary file 1 — Figure S1 [file 41419_2021_3601_MOESM1_ESM.tif]

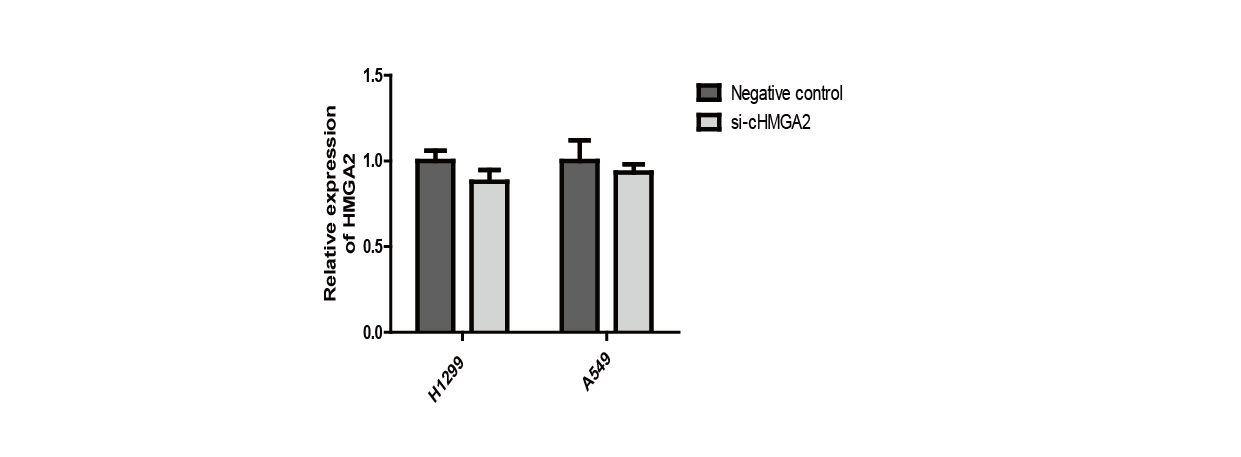

Supplement: Supplementary file 2 — Figure S2 [file 41419_2021_3601_MOESM2_ESM.tif]

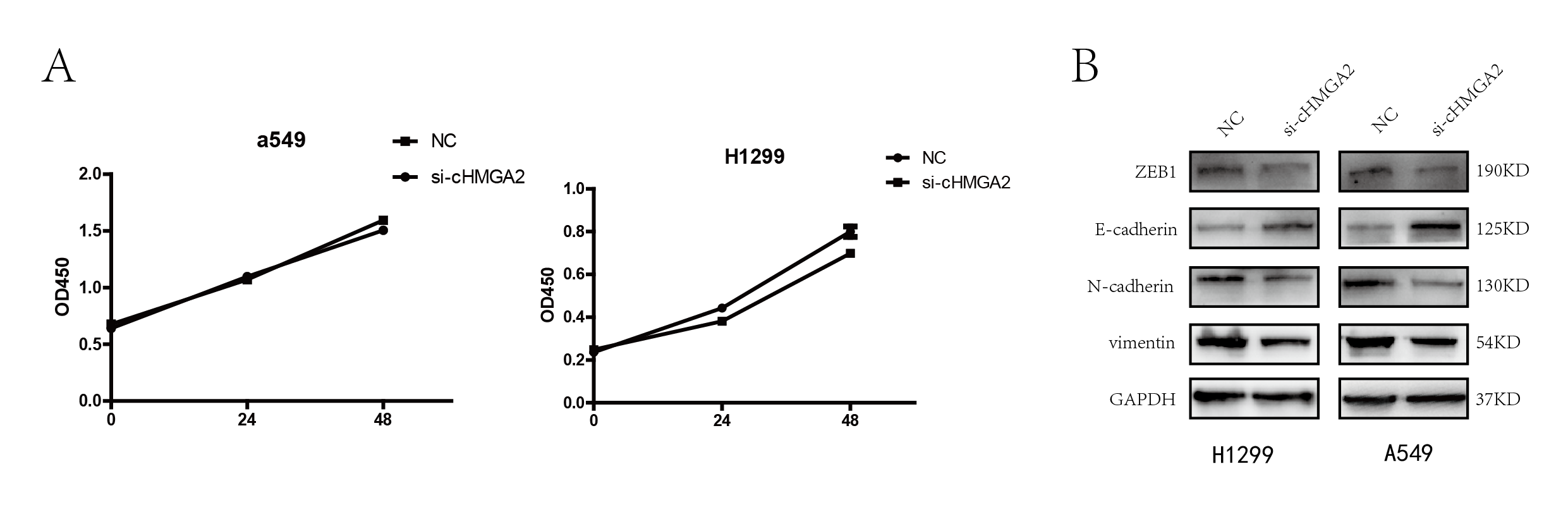

Supplement: Supplementary file 3 — Figure S3 [file 41419_2021_3601_MOESM3_ESM.tif]

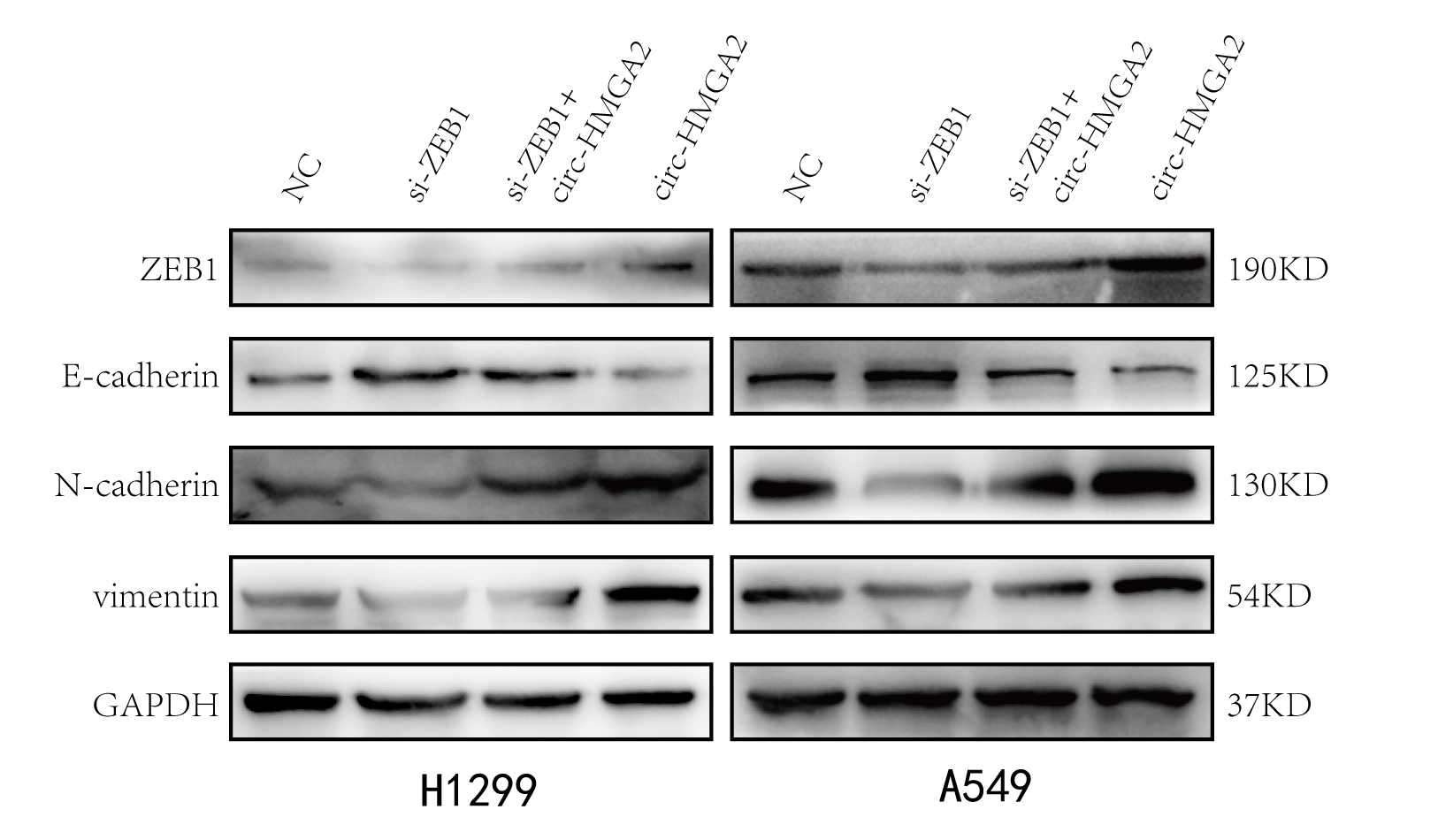

Supplement: Supplementary file 4 — Figure S4 [file 41419_2021_3601_MOESM4_ESM.tif]
