# Supplementary material for: Circ-HMGA2 (hsa_circ_0027446) promotes the metastasis and epithelial-mesenchymal transition of lung adenocarcinoma cells through the miR-1236-3p/ZEB1 axis
Source: Cell Death Dis. 2021 Mar 24;12(4):313. doi: 10.1038/s41419-021-03601-2 (PMC7991034; doi:10.1038/s41419-021-03601-2)
Supplement: Supplementary file 5 — Supplementary Figure Legends [file 41419_2021_3601_MOESM5_ESM.docx]

**Supplementary Material**

Table S1 All primers used in this study

| Gene sequence  GAPDH FORWARD CAGGAGGCATTGCTGATGAT  GAPDH REVERSE GAAGGCTGGGGCTCATTT  ZEB1 FORWARD AAGTGGCGGTAGATGGTAATGT  ZEB1 REVERSE AAGGAAGACTGATGGCTGAAAT  hsa_circ_0027446 FORWARD GCCACTGGAGAAAAACGGCC  hsa_circ_0027446 REVERSE TTGCTGCCTTTGGGTCTTCC  hsa-miR-1236-3p FORWARD GCGCCTCTTCCCCTTGTCT  hsa-miR-1236-3p REVERSE AGTGCAGGGTCCGAGGTATT  hsa-miR-1236-3p RT GTCGTATCCAGTGCAGGGTCCGAGG  TATTCGCACTGGATACGACCTGGAG  U6 FORWARD GCTTCGGCAGCACATATACT  U6 REVERSE GTGCAGGGTCCGAGGTATTC  U6 RT GTTGGCTCTGGTGCAGGGTCCGAGGT  ATTCGCACCAGAGCCAACAAAATATGG |
| --- |

Supporting Fig. S1. (A) Genomic origin of the circRNAs (n = 12,444) identified in human lung tissues and LUAD tissues. (B) Hierarchical clustering showed the circRNAs deregulated in LUAD tissues compared to adjacent normal tissues.

Supporting Fig. S2. Knockdown of circ-HMGA2 expression did not affect the expression of the linear HMGA2 transcript.

Supporting Fig. S3. (A) The results of the CCK8 assay showed that the proliferation of H1299 and A549 cells were not significantly reduced when the expression of circ-HMGA2 was inhibited. (B) It was inhibited the progress of EMT when knocking down the expression of circ-HMGA2 in LUAD cells.

Supporting Fig. S4. Circ-HMGA2 was blocked the effects of si-ZEB1 on EMT
